# Malaria in the Republic of Ireland; A retrospective review of the clinical epidemiology of malaria between 2016 and 2020

**DOI:** 10.1016/j.ijregi.2024.100467

**Published:** 2024-10-11

**Authors:** David Moynan, James O'Connell, Eoghan de Barra

**Affiliations:** 1 Department of Infectious Diseases, Mater Misericordiae University Hospital, Dublin, Ireland; 2 Department of Public Health West, Health Service Executive, Dublin. Ireland; 3 Department of Infectious Diseases, Beaumont Hospital, Dublin, Ireland; 4 Department of Tropical Medicine and International Health, Royal College of Surgeons Ireland, Dublin, Ireland

**Keywords:** Malaria, Imported fever, Public health, Chemoprophylaxis, Tropical medicine, Returning traveler

## Abstract

**Objectives:**

Malaria is a parasitic infection that continues to present significant morbidity and mortality figures throughout the world. In the Republic of Ireland (Ireland), malaria is an imported infection linked most frequently to travel routes from West Africa. There is a paucity of data on the clinical epidemiology and hospital service utilization of malaria in non-endemic setting to inform physician practice. This study aims to fill that gap, providing a clinical context to the national picture of both adult and pediatric cases.

**Methods:**

A retrospective review of data from all public Irish hospitals was performed from 2016 to 2020, inclusive. Ethical approval was granted following review by the Social Research Ethics Committee (SREC) of University College Cork.

**Results and Conclusions:**

During the study period, there were 337 cases of malaria presenting to Irish hospitals, 15.1% (51/337) in the pediatric age category. Presentations occurred nationwide with a predictable seasonality. The majority (77.45%; 261/337) were *Plasmodium falciparum* and 6% (20/337) required critical care admission. Interestingly, females had a higher likelihood of admission to critical care (odds ratio 2.75, 95% confidence interval 1.1-6.86). The COVID-19 pandemic saw a 78% reduction in malaria presentations in 2020. A total of 1,166 bed days were utilized by cases of malaria.

## Highlights

• 15% of all malaria hospitalizations in Ireland are pediatric.
• Female patients are more likely to require critical care for treatment of malaria.
• Malaria hospitalizations utilized 1,166 bed days in the Irish health service from 2016-2020.
• The COVID-19 pandemic saw a reduction in malaria presentations to Irish hospital by 78%.

## Introduction

Malaria is a parasitic infection that continues to present significant morbidity and mortality figures throughout the world [1]. Thought initially to be a disease of “bad air” (*mal'aria*), this infection occupies a unique position in the annals of history and has existed for millennia prior to its identification in 1880 by French physician Charles Louis Alphonse Laveran [2]. Although this infection's sphere of transmission may have shifted throughout history, no region has felt the catastrophic force of malaria quite like Africa, which, in 2019, accounted for 215 million, or 94%, of cases [3]. The large majority of those who die from malaria in Africa are children younger than 5 years, with the high case-fatality rate attributed to late presentation, poor diagnostics, and the limited availability of effective drugs [4].

Transmitted by the *Anopheles* mosquito, the *Plasmodium* parasite is responsible for the disease, with five species known to infect humans: *Plasmodium falciparum, Plasmodium ovale, Plasmodium vivax, Plasmodium malariae*, and *Plasmodium knowlesi* [5]. Infection from *P. falciparum* displays the highest morbidity and mortality and is the dominant species in the African continent [5]. With an estimated 229 million cases of malaria and 409,000 deaths in 2019 globally, this infection remains a key focus for many international organizations, notably the World Health Organization (WHO), which has targeted malaria under its *“Global technical strategy for malaria 2016-2030* [6].” This bold vision has set an ambitious target to reduce global malaria burden by 90% by the year 2030.

In Ireland and throughout most of Europe, malaria is an imported infection linked most frequently to travel routes from West Africa by African migrants visiting friends and relatives (VFR) [7]. Under Ireland's *Infectious Disease Regulations*, malaria is a notifiable disease. For surveillance purposes, it is defined by laboratory criteria through either the demonstration of malarial parasites by light microscopy, the detection of *Plasmodium* nucleic acid in blood, or the detection of the *Plasmodium* antigen [8].

Collaborative data from the Health Protection Surveillance Centre (HPSC) and the European Centre for Disease Prevention and Control (ECDC) place Ireland as the seventh highest on the list for imported malaria in Europe, with a crude incidence of 1.2 cases per 100,000 population in 2018 [8]. Although the number of imported malaria in Ireland may be comparatively high, its rarity within an Irish context risks physician unfamiliarity at point of care. This is of concern, with data from a large study in the United Kingdom (UK) demonstrating increased mortality from *P. falciparum* malaria in the UK regions where the disease is rarely seen [9]. There is a paucity of data on the clinical epidemiology and hospital service utilization of malaria in non-endemic setting to inform physician practice. This study aims to fill that gap, providing a clinical context to the national picture of both adult and pediatric cases.

## Methodology

### Data sources

Data for this study were obtained from the National Quality Assurance and Improvement System (NQAIS) Clinical, an online interactive application that analyses Hospital In-Patient Enquiry (HIPE) data with the aim of providing detailed feedback to clinicians and hospital managers [10]. HIPE records are created based on a patient's episode of care and records diagnostic, procedural, and administrative details. An episode of care is defined as beginning when a patient is admitted to the hospital, as a day case or inpatient, and ends at discharge from (or death in) that hospital [11]. HIPE coders, employed in each of the publicly funded hospitals, record clinical and administrative data from patient records following discharge and assign the appropriate diagnostic-related group code to each record. Of note, within the Irish health care system, 83% of beds are publicly funded, whereas the remainder are located within private for-profit hospitals located around the country [12]. HIPE data is not collected from these private institutions.

A medical card (through the General Medical Services [GMS]) is a non-cash benefit in Ireland that provides free primary, community, and hospital care for those below a specific income means-test threshold [13]. In Ireland, a model four hospital is considered a “hub” for clinical care within a region, with multiple inpatient specialties and a category III intensive care unit (ICU), whereas a model three hospital has a category I or II ICU.

The International Classification of Diseases-10-Australian Modification (ICD-10-AM) 8th Edition is used for coding diagnoses and is based on the WHO ICD-10. To anonymize the dataset, encryption of the patient's Medical Record Number (MRN) is performed to create an E-MRN with the removal of the GMS number, the patient's name, and address and conversion of the patient's date of birth to “age on admission.” Comparative data on national malaria notifications were obtained from the HPSC's infectious disease notifications in Ireland 2014-2019.

### Ethical approval

Ethical approval for this study was granted following review by the Social Research Ethics Committee (SREC) of University College Cork. Permission to use data from NQAIS Clinical was authorized by both the National Clinical Lead for the Acute Medicine Division of the Health Service Executive (HSE) and the National Clinical Advisor and Group Lead for the Acute Hospitals Division of the HSE.

### Data extraction

The NQAIS Clinical was searched for all episodes of care due to malaria, where the discharge/death occurred between January 01, 2016 and December 31, 2020. An episode of care due to malaria was defined as an admission with an ICD-10 code for malaria (B500, B508, B509, B510, B518, B519, B520, B528, B529, B530, B531, B538, B54) as the principal diagnosis (see Appendix 1.). For every episode of care, variables were extracted, including hospital name, admission type (emergency/elective), hospital transfer (yes/no), admission date, age on admission, length of stay (LOS) (days), sex, high dependency unit [HDU]/ICU) bed required (yes/no), primary medical specialty supervising care, and Charlson comorbidity index, a validated tool used to quantify the impact that a set of specific comorbidities have on a patient's survival [10,14,15]. The NQAIS Clinical dataset does not contain information pertaining to patient's travel history and presumed risk factors for disease acquisition.

### Data analysis

The dataset was screened for duplication and matching was performed on all episodes of care by comparing age, sex, county of residence, discharge destination, and the E-MRNs extracted from NQAIS Clinical.

Statistical analysis was performed using Stata/SE version 16.1 (StataCorp 2019) for Mac. A two-sample *t*-test was used to compare the LOS between primary supervising teams. A logistic regression analysis was performed to understand the effects of age, sex, and the patient's Charlson score on admission to an HDU/ICU. A *P*-value of ≤0.05 was used to represent statistical significance.

## Results

### Patient characteristics

During the 5-year period between January 01, 2016 and December 31, 2020, there were 363 episodes of care with malaria identified at the principal diagnosis in the Irish public health care system. After a data-matching process, 26 episodes of care were considered duplicates and excluded from the analysis, with n = 337 as the final study population.

The median age of this cohort was 38 years (interquartile range 21-55 years). At the time of admission, 51/337 (15.1%) were younger than 16 years and 228/337 (67.6%) were male. Most of the patients in this study had no significant past medical history, with 312/337 (92.5%) having a Charlson comorbidity index of 0. This suggests that the vast majority of patients did not have any of the comorbidities that are weighted in the scoring tool, including diabetes mellitus, liver disease, and HIV, among others [14]. The remainder (7.5%) of patients were noted to have comorbidities of varying degrees, with one patient scoring 21, reflecting significant comorbidities. Almost half of all patients (45.4%) were medical card holders.

### Episode of care characteristics

Summary statistics relating to each episode of care are outlined in Table 1. Almost all malaria hospitalizations (335/337, 99.4%) were emergency admissions, with only two documented as elective. A hospital-to-hospital transfer was required in 11/337 (3.26%) of patients. These were all from model three hospitals to model four hospitals, suggesting a greater need for higher specialist and critical care. An inpatient infectious diseases team (including genitourinary medicine, pediatric infectious diseases, and adult infectious diseases) was specified as the primary medical service for 90/337 (26.7%) of all hospitalizations. The median LOS was 3 days (interquartile range 1-5 days) with a total of 1,166 bed days utilized throughout the 5-year period, 170.5 bed days among the pediatric age cohort, and 945.5 days among the adult age cohort.

**Table 1:** Summary statistics of malaria episodes of care between 2016 and 2020.

Summary statistics	
Patient characteristics	Median (interquartile range) or N (%)
Total episodes	337
Median age of patients	38 (21-55)
Medical card holder	153 (45.4%)
Pediatric admission^a^	51 (15.1%)
Male	228 (67.6%)
CCI = 0	312 (92.5%)
CCI >0	25 (7.5%)
CCI 2	2 (0.6%)
3	16 (4.75%)
4	2 (0.59%)
10	1 (0.3%)
11	1 (0.3%)
13	2 (0.6%)
21	1 (0.3%)
Episode of care characteristics	
Emergency admission	335 (99.4%)
Hospital-hospital transfers	11 (3.26%)
Admission under infectious diseases service^b^	90 (26.7%)
HDU/ICU care required	20 (6%)
Death	1 (0.3%)
Same day discharge	22 (6.5%)
Length of stay	3 (interquartile range 1-5)
Total bed days used	1166
Pediatric (mean)	170.5 (3.34)
Adult (mean)	945.5 (3.48)
HDU/ICU bed days used	85
Pediatric	12
Adult	73

CCI, Charlson comorbidity index; HDU/ICU, high dependency unit/intensive care unit.

a Pediatric admissions classified as under 16 years of age in the Republic of Ireland.

b Including pediatric infectious diseases, adult infectious diseases and genitourinary medicine.

The mean LOS under an infectious diseases inpatient service was 3.8 days (SD 6.5, 95% confidence interval [CI] 3.15-4.52), whereas that under all other services was 3.3 days (SD 2.71, 95% CI 3.13-3.51). This difference was not statistically significant (*P* = 0.054). The mean LOS for a pediatric patient, 3.3 days (SD 1.97, 95% CI 3.04-3.63), was not different from the mean adult LOS of 3.48 days (SD 4.35, 95% CI 3.20-3.75) (*P* = 0.68). A same-day discharge, a discharge within 24 hours of hospital admission, was documented in 22/337 (6.5%) of cases. An infectious diseases inpatient service accounted for 6/22 (27.3%) of these same-day discharges.

**Figure 1:**
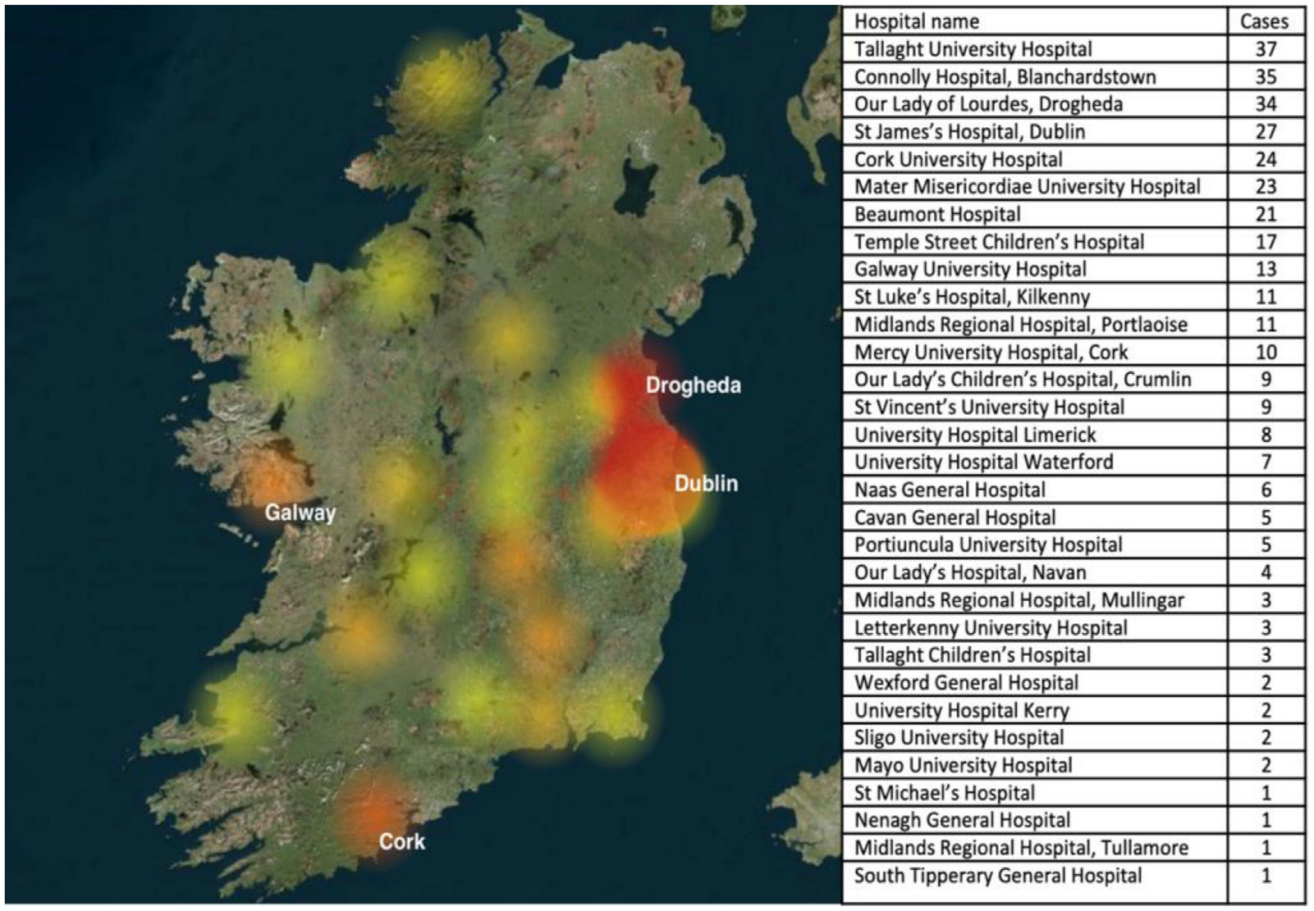
Heat map demonstrating the hospitals of the Republic of Ireland with the highest frequency of malaria hospitalizations (red) and the lowest frequency (yellow). Associated hospitalizations as per hospital are listed in order of frequency.

Among the cohort, 20/337 (6%) required HDU/ICU care. A total of 85 inpatient bed days were utilized in HDU/ICU throughout this 5-year period. There is evidence of the association between female sex and admission to the HDU/ICU with malaria, with an odds ratio (OR) of 2.75 (95% CI 1.10-6.86, *P* = 0.03). Although pregnancy status is not a variable routinely collected in the HIPE dataset, two patients were documented as pregnant, both of whom required HDU/ICU care. Figure 1 demonstrates the wide distribution and frequency of malaria admissions to Irish hospitals throughout the country.

Although age was not found to be associated with an increased risk of HDU/ICU admission as per Table 2, having a Charlson score of greater than 0 increases the risk of admission (OR = 1.79, 95% CI 0.37-8.60; *P* = 0.47). Within this 5-year period, there was one reported death from malaria in a person with a Charlson score of zero. This death occurred on the third day of admission outside of the HDU/ICU setting.

**Table 2:** Admission to high dependency unit/intensive care unit across age, Charlson comorbidity index (greater than 0), and sex.

	N admitted	Odds ratio	95% confidence interval	*P*-value
Age	20	0.99	0.97-1.02	0.71
Charlson score				
Score 0 (reference)	10	1 (reference)	0.33-7.57	0.56
Score >0	2	1.59		
Sex				
Male	9	1 (reference)	1.10-6.86	0.03
Female	11	2.75		

Data from the HPSC demonstrate that *P. falciparum* is the dominant species of malaria identified in Ireland, accounting for 83% of infections in 2018 [16]. This is consistent with data extracted from NQAIS Clinical, with 261/337 (77.45%) of all cases of malaria admitted to hospital identified as *P. falciparum*, 16/337 (4.75%) *P. ovale*, 10/337 (3%) *P. vivax*, 3/337 (0.9%) *P. malariae*, and 47/337 (14%) unspecified. Examining the pediatric cohort in particular, 42/51 (82.4%) of admissions were related to *P. falciparum*, 3/51 (5.9%) *P. ovale*, 1/52 (2%) *P. malariae*, and 5/51 (9.8%) unspecified.

**Figure 2:**
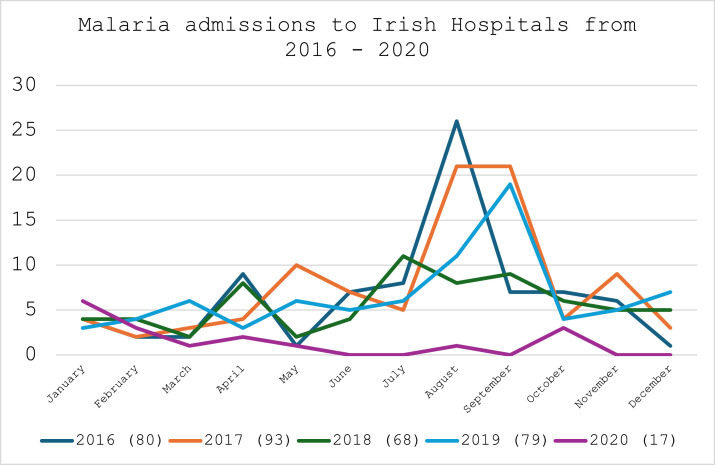
Trend graph illustrating the monthly variation in malaria admissions to hospital from 2016 to 2020.

The HPSC, in its report on the burden of imported malaria in Ireland in 2010, identified a significant seasonality associated with malaria in Ireland with the highest case number in the third quarter of the year [17]. This is again consistent with data from NQAIS Clinical, which highlights a two- to four-fold increase in malaria hospitalizations in the months of July, August, and September (Figure 2). Interestingly, with the advent of the COVID-19 pandemic, malaria hospitalizations fell significantly in 2020, with only 17 cases identified, compared with an average of 80 malaria hospitalizations per year in the previous 4 years.

## Discussion

This study demonstrates that malaria, a disease not endemic to Ireland, presents in significant numbers throughout the country in both urban and rural hospital settings, also noted in other European countries. The HPSC's publication on malaria trends in Ireland highlights that the majority of malaria cases are acquired when traveling to endemic regions VFR, most commonly in Western Africa [8]. Data from 2018 note that 65% of all cases of malaria in Ireland arose in the VFR group, 59% of which were from Nigeria alone, reflecting Ireland's importance as a destination for those emigrating from English-speaking West Africa [8]. With 77.45% of all malaria cases in the last 5 years identified as *P. falciparum* from NQAIS Clinical data, the offending parasite is reflective of the dominant African region of exposure of our cohort. These figures are concerning, however, as malaria infection is largely preventable with the use of prophylactic anti-malarial medications and mosquito nets [18]. This raises the question of traveler awareness regarding the perceived risk of infection. Although those who reside in areas of high endemicity are exposed to levels of parasitemia that may confer clinical immunity, this can be lost within 3-5 years without re-exposure, with semi-immunity lost after 6 months [19]. Furthermore, 15.1% of all malaria hospitalizations in the last 5 years have been pediatric (<16 years of age). Assuming these children have been raised in Ireland, it is likely they will have no immunity to malaria and are particularly vulnerable to infection [19]. As such, much of the African VFR population may not be aware of the significant risk of malaria posed to them on traveling.

There have been studies examining the attitudes of the African VFR population towards malaria infection and their self-perceived risk of disease. An Irish study noted a lack of awareness of critical aspects of malaria transmission and prevention among an African VFR population in the west of Ireland [20]. In this paper, 17% of those questioned believed they were not at risk of contracting malaria when returning to Africa, whereas 28% did not believe malaria to be a serious illness. However, there is likely a heterogeneity among the barriers to malaria prevention in VFR groups, with education on chemoprophylaxis alone unlikely to result in a significant uptake in adherence when traveling overseas [21]. With many VFR groups underestimating the dangers of malaria, communicating its severity and the high efficacy of chemoprophylaxis and mosquito nets is integral to ensuring the uptake of these primary preventative measures.

Health care cost as a barrier to the uptake of primary preventative measures against malaria is likely impacting the use of chemoprophylaxis further [22]. The data shows that only 45.4% of those hospitalized were in receipt of a medical card; as such, attending primary care for a prescription and the subsequent procurement of chemoprophylaxis is not funded by the Irish State. This barrier has been lifted somewhat in the UK, with the availability of atovaquone/proguanil without prescription in community pharmacies [23]. The authors of this initiative argue that the availability of a non-priority service such as travel medicine may become more limited in the context of increasing demands on our primary care services and a community pharmacy initiative, with the correct governance, can widen accessibility for travelers [23].

There is a predictable seasonality associated with imported malaria in Ireland, as evidenced in Figure 1. This peak of malaria cases in the third quarter of the year coincides with the wet season in West Africa, the period between May and October, when mosquito density has been shown to be the highest [24]. However, results from 2020 show a significant decrease in the numbers of malaria, with only 17 episodes of care reported in NQAIS Clinical. This is down 78% from 2019 figures, which stood at 79 episodes of care. The global pandemic from COVID-19 and the subsequent dramatic decline in overseas departures and arrivals is certainly the driver behind this reduction, with overseas arrivals down 78% in 2020 compared with 2019 [25].

Malaria has, in the last 5 years, utilized significant resources from within the Irish public health care system. The Healthcare Pricing Office (HPO) within the HSE categorizes hospital admissions according to a specified diagnosis-related grouping. Each grouping is based on cases that are clinically similar and typically consume the same amount of resources [26]. According to data from 2020, the inpatient inlier price for a malaria hospitalization ranges from € 3920 to € 19,640 depending on the level of complexity as determined by diagnosis-related grouping coding, of which there are three [27,28]. Data from Public Health England suggests that the cost of chemoprophylaxis may be a deterrent for many traveling to at-risk areas [29]; as such, an official cost analysis on the provision of malaria chemoprophylaxis for VFRs would be welcomed.

Research from the UK has identified both a presentation to a health care facility in the UK where malaria is rarely seen and older age as significant risk factors for mortality in *P. falciparum* malaria [9]. In this study, while logistic regression failed to identify a significant association between age and admission to HDU/ICU (a marker of severe illness), there was a statistically significant association between female sex and admission to HDU/ICU (OR 2.6, 95% CI 1.03-6.6, *P* = 0.04). Although pregnancy status is not routinely collected through HIPE data, two female patients were documented as pregnant and both required HDU/ICU care. This is consistent with existing data that shows pregnancy as a risk for severe malaria and unfavorable outcomes, including death [30]. Interestingly, an increased risk for severe *P. falciparum* malaria has previously been reported among non-pregnant females compared with males, though no clear physiologic rationale has been identified [31].

Importantly, presenting to a health care facility that has a low frequency of malaria hospitalizations risks physician unfamiliarity and may lead to delays in diagnosis and subsequent treatment errors [9,32]. Obtaining a detailed travel history, a skill that allows physicians to remain alert to the potential for tropical diseases is not being adequately performed. One Irish study noted that only 25% of ill returning travelers were questioned about pre-travel vaccinations and only 57% questioned regarding malaria chemoprophylaxis [33]. In addition, treatment errors continue to occur with regard to malaria management [32]. Acknowledging this as a contributor to poor patient care, an acute teaching hospital in Dublin introduced interventions to mitigate against malaria treatment errors at their institution, focusing on staff education, the reconfiguration of existing hospital guidelines, and enabling rapid access to medications for staff providing care to patients. This has also been emphasized at a European level, with the European Society for Clinical Microbiology and Infectious Diseases (ESCMID) study group on parasitology noting the importance of an institution's access to experienced microscopy for diagnostic purposes and adequate therapy, specifically intravenous artesunate for severe or complicated disease [34]. As such, throughout Europe, maintaining familiarity with the clinical presentation, diagnosis, and management of malaria is paramount to ensure that good clinical care is provided to those who present to our hospitals.

The strengths of this study include its collection of data on malaria admissions to all acute public hospitals throughout the country, including pediatric hospitals. This allows for a complete narrative on malaria for both adults and pediatric patients; however, it is limited by several factors. First, admissions to Ireland's private hospitals are not captured by HIPE data and, as such, are not represented by NQAIS Clinical. Furthermore, given Ireland's lack of a universal patient identifier, there is potential for duplicates within the dataset, although a rigorous matching process was completed prior to data analysis. Importantly, despite the importance of VFR groups on Ireland's malaria numbers, this dataset does not examine the country of origin, nor does it contain information regarding the use of chemoprophylaxis or the reason for travel. However, this data is typically published separately by the HPSC in their annual malaria reports, as noted above.

Future work on imported malaria in European countries must examine barriers to chemoprophylaxis among VFR groups, including the cost of medicines and exploring the understanding of therapeutic efficacy among those who travel to endemic regions. Examining the impact of health care costs on primary prevention and addressing upstream barrier reduction strategies that might make access to health care more achievable are integral to reducing the morbidity, mortality, and health care cost of imported malaria in Ireland.

## Conclusion

To our knowledge, this is the largest study dedicated to the clinical epidemiology of imported malaria in both the adult and pediatric populations in Ireland. Data from this study provides a clinical context to the statistics available from the HPSC and highlights the significant use of resources, both monetary and acute-bed usage, attributed to malaria in the health service. From a public health perspective, this study emphasizes not only a greater need for education and access to malaria chemoprophylaxis for the VFR group and other tourists but also the need for appropriate training on imported fevers for frontline health care workers, in particular during the third quarter of the year when rates of imported malaria are highest.

## CRediT authorship contribution statement

**David Moynan:** Conceptualization, Data curation, Investigation, Formal analysis, Writing – original draft, Writing – review & editing. **James O'Connell:** Data curation, Writing – review & editing. **Eoghan de Barra:** Writing – review & editing, Supervision.

## Declarations of competing interest

The authors have no competing interests to declare.

## Figures and Tables

**Table:** 

**B500**	*Plasmodium falciparum* malaria with cerebral complications	**B528**	*Plasmodium malariae* malaria with other complications
**B508**	Other severe and complicated *Plasmodium falciparum* malaria	**B529**	*Plasmodium malariae* malaria without complications
**B509**	*Plasmodium falciparum* malaria, unspecified	**B530**	*Plasmodium ovale* malaria
**B510**	*Plasmodium vivax* malaria with rupture of spleen	**B531**	Malaria due to simian Plasmodia
**B518**	*Plasmodium vivax* malaria with other complications	**B538**	Other malaria, not elsewhere specified
**B519**	*Plasmodium vivax* malaria without complications	**B54**	Unspecified malaria
**B520**	*Plasmodium malariae* malaria with nephropathy		
